# Targeting FAP/CAFs to rewire immune-excluded and resistant tumor niches

**DOI:** 10.3389/fphar.2026.1812674

**Published:** 2026-06-17

**Authors:** Yingying Wang, Yajun Zhao, Ce Zhou, Yang Wang

**Affiliations:** Department of Rehabilitation, Qingdao Municipal Hospital, Qingdao, China

**Keywords:** cancer-associated fibroblasts, checkpoint blockade resistance, FAPI-PET, fibroblast activation protein, immune exclusion, tumor microenvironment

## Abstract

Immune checkpoint blockade (ICB) has reshaped cancer therapy, yet many solid tumors remain immune-excluded, with cytotoxic T cells trapped in stromal regions and unable to access malignant nests. In these settings, cancer-associated fibroblasts (CAFs) and their extracellular matrix programs act as active ecosystem regulators that impose physical transport barriers, chemokine “gating” (notably CXCL12–CXCR4), and stromal exclusion signals such as TGF-β, collectively sustaining resistance to both immunotherapy and targeted agents, and contributing to treatment intolerance and rehabilitation-relevant functional burden. Building on this mechanistic blueprint, we review clinically relevant strategies that use fibroblast activation protein (FAP), when sufficiently expressed and spatially relevant, as a tractable stromal address label to rewire resistant niches, ranging from FAP-targeted immunocytokines and conditional costimulation to regional FAP-CAR-T approaches and FAPI-PET–enabled stratification/theranostics. We highlight key safety constraints and failure modes that can limit not only efficacy but also functional recovery, and propose a trial-ready roadmap centered on state-guided selection, mechanism-matched combinations, early pharmacodynamic verification of rewiring, and pragmatic functional endpoints.

## 1 Introduction

Despite the success of ICB, immune-excluded tumors remain a major clinical challenge because antitumor T cells may be present but spatially separated from malignant nests. This spatially constrained immunotype—often discussed alongside “inflamed” and “desert” states—has become a practical framework for understanding differential response to immunotherapy and for designing mechanism-guided combinations (Mellman et al., 2023; Roerden and Spranger, 2025; Tiwari et al., 2023; Kather et al., 2018). A central and increasingly actionable driver of immune exclusion is the CAF–extracellular matrix (ECM) axis. CAFs are not merely structural bystanders: they remodel ECM, regulate tissue mechanics and interstitial pressure, and shape chemokine/cytokine gradients that govern immune trafficking. In parallel, these stromal programs contribute to clinically meaningful symptom and function burdens, such as pain, stiffness, fatigue, and reduced physical capacity, thereby affecting treatment tolerance and the feasibility of timely rehabilitation during active therapy and survivorship. Embedding pragmatic functional endpoints (physical function or mobility measures) alongside ecosystem-state biomarkers can help align niche-rewiring strategies with rehabilitation-oriented care (Silver et al., 2013; Stout et al., 2021; Silver et al., 2015). Importantly, CAFs are heterogeneous, with context-dependent tumor-promoting or tumor-restraining functions, helping to explain why “blanket stromal depletion” has produced mixed results and why state-aware, mechanism-specific targeting is now favored (Sahai et al., 2020; P et al., 2026; Estrach et al., 2024). In particular, matrix-remodeling myCAF states, cytokine/chemokine-producing iCAF states, and immune-interacting apCAF-like states may contribute to immune exclusion through different mechanisms and may therefore require different therapeutic approaches.

Within this stromal ecosystem, FAP stands out as a clinically tractable surface marker enriched on activated fibroblasts in many carcinomas and desmoplastic tumors. However, FAP should not be interpreted as a universal CAF marker or a tumor-specific antigen. Its expression varies across tumor types, disease stages, metastatic sites, and prior treatment contexts. In addition, FAP can be induced in non-malignant tissue-remodeling conditions, including wound healing, fibrosis, and chronic inflammation. Therefore, FAP is best viewed as a context-dependent stromal address label that requires confirmation by imaging, histology, or spatial profiling before being used for patient selection or therapeutic delivery. Early clinical attempts to target FAP with the humanized monoclonal antibody sibrotuzumab (F19) demonstrated feasibility and safety but limited antitumor activity, underscoring that FAP targeting must be coupled to an appropriate payload, combination partner, and patient-selection strategy to translate into meaningful niche rewiring (Hofheinz et al., 2003; Scott et al., 2003; Wu et al., 2025). The field is now shifting toward rewiring immune-excluded and resistant tumor niches rather than simply removing stroma. Clinical translation is emerging along three complementary routes: (i) CAF/ECM barrier modulation, exemplified by hyaluronan-targeted stromal remodeling in hyaluronan-high pancreatic cancer; (ii) blocking stromal exclusion programs, such as TGF-β-driven immune evasion; and (iii) FAP-targeted delivery of immune activation, including FAP-directed cytokine or costimulatory platforms designed to concentrate immune stimulation within FAP-rich tumor stroma while limiting systemic exposure (Van Cutsem et al., 2020; Tauriello et al., 2022; Melero et al., 2023; Koyama et al., 2024; Suarez-Carmona et al., 2021).

**FIGURE 1:**
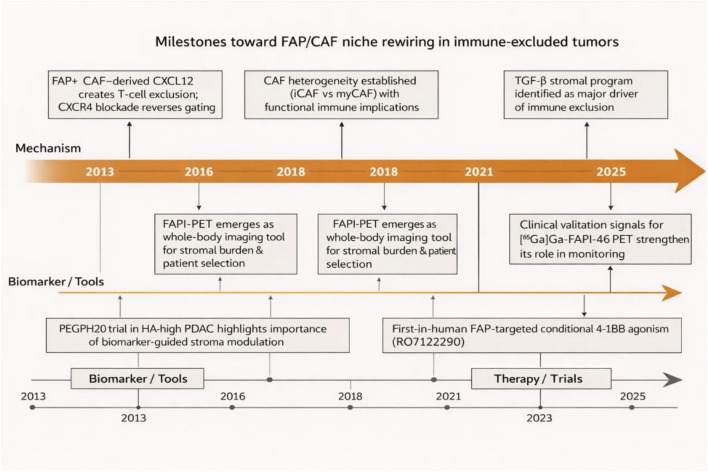
Milestones and decision points toward FAP/CAF niche rewiring (2013–2025). Beyond summarizing chronological advances, the timeline maps key discoveries onto therapy-design decisions. CXCL12–CXCR4 signaling, ECM/HA-associated transport restriction, and TGF-β-driven stromal signaling represent interacting exclusion nodes. CAF heterogeneity and plasticity explain why broad stromal depletion may fail and why state-specific rewiring is preferred. FAPI-PET, spatial immune profiling, and early pharmacodynamic readouts then provide tools for patient selection and target-engagement verification. Together, these milestones support a decision sequence of defining the dominant stromal state, selecting a matched intervention, and confirming early biological remodeling.

To contextualize these developments, Figure 1 summarizes the chronological evolution from mechanistic discoveries to therapy-design principles. Rather than serving only as a timeline, the figure highlights three decision nodes for CAF/FAP-directed strategies: identifying the dominant exclusion mechanism, selecting a matched therapeutic lever, and verifying early pharmacodynamic target engagement. This framework emphasizes that successful translation requires state-guided patient selection (such as stromal burden and immune-excluded architecture), mechanism-matched combinations, and early pharmacodynamic confirmation of intratumoral immune access. In this mini-review, we synthesize how FAP/CAF-directed strategies can convert immune-excluded, therapy-resistant niches into more permissive ecosystems, with an emphasis on clinically relevant modalities, safety constraints, and practical stratification concepts that enable rational combination design.

## 2 Mechanistic basis of FAP/CAF-driven immune exclusion and resistance

### 2.1 ECM mechanics: matrix density, solid stress, and impaired transport

Immune-excluded, stroma-rich tumors often behave like transport-limited tissues: excessive matrix deposition and remodeling raise interstitial fluid pressure and solid stress, compress microvessels, and reduce effective perfusion, collectively restricting both drug penetration and T-cell migration. A mechanistic literature anchored in tumor biophysics frames this as a coupled “solid + fluid” problem in which mechanical forces and abnormal ECM architecture directly limit therapeutic delivery (Jain et al., 2014; Stylianopoulos et al., 2018; Stylianopoulos et al., 2012). Clinically, these transport-limited stromal states often co-occur with higher symptom burden and reduced physical function, which can compromise treatment adherence and narrow the window for timely rehabilitation during active therapy (Fletcher and Johnson, 2024; Ligibel et al., 2022). This logic motivated hyaluronan (HA) depletion as a stromal remodeling strategy in pancreatic ductal adenocarcinoma (PDAC). In the phase III HALO-301 trial, Pegvorhyaluronidase Alfa (PEGPH20) was tested in HA-high metastatic PDAC to degrade HA and remodel the stroma, providing an instructive clinical test of whether matrix decompression can improve outcomes in a biomarker-selected setting. In HALO-301, PEGPH20 plus nab-paclitaxel/gemcitabine did not improve survival despite biomarker enrichment for HA-high metastatic PDAC: median overall survival was 11.2 versus 11.5 months and median progression-free survival was 7.1 versus 7.1 months, although the objective response rate was numerically higher with PEGPH20-containing therapy. This disconnect between response and survival highlights that radiographic shrinkage or transient stromal decompression may not be sufficient to durably rewire an immune-excluded ecosystem (Van Cutsem et al., 2020).

The failure of HALO-301 should therefore be interpreted as an informative failure of stromal pharmacology rather than as definitive evidence that CAF/ECM targeting is biologically irrelevant. Several factors may have contributed (Hakim et al., 2019). First, HA-high status may have been an incomplete enrichment biomarker. Although HA accumulation reflects one component of a transport-limited microenvironment, it does not necessarily identify tumors in which HA is the dominant driver of immune exclusion, drug resistance, or poor perfusion. Matrix architecture, vascular compression, CAF subtype composition, myeloid inflammation, and TGF-β activity may all modify whether HA depletion translates into therapeutic benefit. Second, enzymatic matrix depletion may transiently decompress the tumor but may not durably reverse chemokine- or cytokine-driven exclusion programs unless paired with an appropriately timed immune-activating or checkpoint-based strategy. Third, stromal perturbation can provoke compensatory rebound, including renewed ECM deposition, inflammatory fibroblast activation, myeloid recruitment, or alternative stromal programs that restore immune exclusion. Fourth, toxicity and timing are critical. In advanced metastatic PDAC, patients often have limited physiological reserve, and platform-specific adverse events, treatment interruptions, or late intervention in an already highly remodeled tumor ecosystem may narrow the therapeutic window (De Jesus-Acosta et al., 2020). Therefore, HALO-301 argues against single-biomarker, single-mechanism stromal depletion and instead supports multidimensional selection, mechanism-matched combinations, and early pharmacodynamic confirmation of stromal rewiring.

### 2.2 CXCL12–CXCR4 axis: stromal retention and mislocalization of T cells

A particularly direct link between FAP^+^ CAFs and T-cell mislocalization is the CXCL12–CXCR4 axis. In a landmark PDAC study, CXCL12 produced by FAP^+^ carcinoma-associated fibroblasts was shown to orchestrate immune evasion, and pharmacologic CXCR4 blockade could rapidly increase intratumoral T-cell access and sensitize tumors to checkpoint blockade in preclinical models (Feig et al., 2013). This axis has also been pushed toward clinical translation. In a phase I/II setting, NOX-A12 (anti-CXCL12) was used as a priming agent followed by pembrolizumab in metastatic colorectal and pancreatic cancer (OPERA), with evidence of microenvironment modulation and disease stabilization in subsets, including disease stabilization in approximately 27% of colorectal cancer and 22% of pancreatic cancer patients, although objective responses remained limited (Suarez-Carmona et al., 2021).

Complementary clinical and translational work has examined CXCR4 inhibition strategies as well, reinforcing that the CXCL12–CXCR4 circuit is a realistic, druggable stromal checkpoint but one that may trigger compensatory programs, such as myeloid shifts, that need to be anticipated in combination design (Chen et al., 2019; Meng et al., 2025).

### 2.3 TGF-β signaling: stabilization of fibroblast activation and immune suppression

Beyond chemokines, TGF-β signaling in stromal cells has emerged as a canonical molecular program associated with immune-excluded tumors. Analyses of human tumor samples linked a fibroblast TGF-β response signature with non-response to PD-L1 blockade, and mechanistic experiments demonstrated that co-inhibition of TGF-β and PD-L1 can convert excluded tumors toward a more inflamed architecture by enabling T-cell penetration (Mariathasan et al., 2018). These findings indicate that stromal signaling can be the dominant limiter of PD-L1 efficacy, even when the therapeutic target is nominally immune-cell intrinsic.

### 2.4 An integrated systems model of CAF-driven immune exclusion

These mechanisms should not be viewed as independent barriers. In immune-excluded tumors, ECM remodeling, chemokine gating, and TGF-β signaling often form a self-reinforcing stromal circuit. ECM deposition and matrix crosslinking increase tissue stiffness, solid stress, and interstitial pressure, thereby compressing vessels, reducing perfusion, and limiting both drug delivery and T-cell migration. In parallel, FAP^+^ CAF-derived CXCL12 and related chemokines retain T cells in stromal compartments or at the tumor margin, preventing productive contact with malignant cells. TGF-β further stabilizes this excluded state by promoting fibroblast activation, ECM production, immune suppression, and impaired effector-cell penetration (Bruni et al., 2023; Melssen et al., 2023). Together, these processes create a three-layer exclusion architecture: a mechanical barrier that limits transport, a chemokine barrier that misdirects immune-cell trafficking, and a signaling barrier that suppresses immune activation (Xiao et al., 2023). This systems-level model explains why single-agent stromal interventions often underperform and why rational combinations should be selected according to the dominant barrier in each tumor ecosystem.

### 2.5 CAF heterogeneity, spatial organization, and plasticity: why rewiring must be state-aware

A key lesson since 2019 is that CAFs are not one entity: single-cell and functional studies in PDAC identified distinct CAF states (inflammatory CAFs vs. myofibroblastic CAFs) with different spatial distributions and immunologic consequences, and later work further expanded CAF diversity (including antigen-presenting-like CAF programs) (Öhlund et al., 2017; Yamashita and Kumamoto, 2024). This heterogeneity helps explain mixed clinical outcomes from broad stromal interventions: removing or suppressing one stromal component can provoke ecosystem rebound (alternative fibroblast programs, myeloid recruitment, or ECM re-stiffening), and in some contexts may eliminate tumor-restraining fibroblast functions. Practically, it argues that effective niche rewiring should pair (i) a clearly defined stromal mechanism (barrier relief vs. chemokine gating vs. stromal cytokine signaling) with (ii) a measurable state classifier (such as HA-high, TGF-β-high stroma, CXCL12/CXCR4-high gating, FAP-high fibroblast burden), so that CAF/FAP targeting becomes a stratified, testable pharmacology rather than a one-size-fits-all concept (Van Cutsem et al., 2020).

CAF heterogeneity can be operationally simplified into several functional states with distinct implications for immune exclusion and therapy. Myofibroblastic CAFs (myCAFs) are enriched in contractile and ECM-remodeling programs, such as α-SMA expression, collagen deposition, and matrix stiffening, and are therefore closely linked to solid stress, impaired perfusion, and physical restriction of T-cell entry. Inflammatory CAFs (iCAFs) produce cytokines and chemokines, including IL-6 and CXCL12, which can promote immune-cell mislocalization, myeloid recruitment, and resistance to checkpoint blockade. Antigen-presenting CAF-like cells (apCAFs) express MHC class II-related programs and may interact with T cells, but their incomplete costimulatory capacity may contribute to dysfunctional or tolerogenic immune interactions. These CAF states also show spatial organization: myCAF-like programs often accumulate near malignant nests, whereas iCAF-like programs may occupy more distal stromal regions and establish soluble exclusion fields. Importantly, CAF states are plastic rather than fixed; therapy, inflammation, hypoxia, and mechanical stress can shift CAF phenotypes and drive stromal rebound. Therefore, CAF/FAP-directed therapy should be framed as state-specific rewiring rather than universal CAF depletion, with the dominant CAF program guiding whether the preferred strategy is matrix normalization, chemokine/cytokine blockade, immune costimulation, or local stromal reprogramming (Elyada et al., 2019).

## 3 Clinical toolbox for FAP and CAF niche rewiring

The clinical maturity of CAF/FAP-directed strategies varies substantially across modalities. HA depletion has been tested in a randomized phase III setting, whereas CXCL12–CXCR4 blockade, FAP-targeted immunocytokines, conditional costimulation, FAP-CAR-T, and FAP radioligand approaches are mostly supported by phase I/II, early theranostic, or preclinical/translational evidence. Therefore, apparent differences in efficacy should not be interpreted as direct cross-strategy comparisons, because tumor type, treatment line, patient selection, combination backbone, and endpoint maturity differ substantially across studies. A clinically oriented toolbox summarizing the major CAF/FAP niche-rewiring routes, therapeutic levers, representative modalities, selection readouts, and key risks is provided in Table 1.

**TABLE 1:** Clinically oriented toolbox for FAP/CAF niche rewiring in immune-excluded, resistant tumors.

Rewiring route	Therapeutic lever	Representative modality	Typical use-case context	Practical PD/Selection readouts	Main risks/Failure modes
Barrier relief	ECM density/ solid stress/ IFP	Stromal remodeling; HA depletion; anti-fibrotic decompression	Desmoplastic, poorly perfused tumors; PDAC-like stroma	Perfusion/drug delivery; HA/ECM markers; intratumoral CD8 distribution	State mismatch; edema/thromboembolic risk; stromal rebound
Chemokine gate release	CXCL12–CXCR4-mediated T-cell retention	CXCL12 neutralization; CXCR4 blockade	Stromal T-cell trapping; CXCL12-rich tumors	Rim-to-core CD8 shift; CXCL12 signaling; inflammatory chemokines	Transient effect; myeloid rebound; systemic immune effects
Exclusion program blockade	TGF-β–driven stromal suppression	TGF-β inhibitors; antibodies; traps	TGF-β-high stromal tumors	pSMAD/TGF-β program; antigen presentation; CD8 access	Narrow therapeutic window; off-tumor effects; incomplete reversal
FAP-targeted immune activation	Local immune stimulation in FAP^+^ stroma	FAP-directed immunocytokines; IL-2 variant	FAP-high tumors; limited systemic cytokine tolerance	Immune activation signatures; intratumoral T cells; cytokine markers	Cytokine-class AEs; FAP density/accessibility dependence
FAP-targeted conditional costimulation	Tumor-localized costimulation	FAP-anchored conditional agonists; 4-1BB platforms	T-cell boosting with limited systemic stimulation	T-cell activation/IFN-γ programs; FAP–T-cell co-localization	On-target/off-tumor activation; liver/immune toxicities; state mismatch
FAP-directed cellular therapy	Local FAP^+^ stromal depletion/reprogramming	Regional FAP-CAR-T; local delivery	Locoregional disease; high systemic-risk settings	Stromal remodeling; immune cell-penetration; imaging/biopsy confirmation	On-target/off-tumor toxicity; local inflammation; antigen heterogeneity
Patient selection and monitoring tool	Stromal burden and treatment engagement	FAPI-PET for selection/monitoring; theranostic	FAP-high selection; longitudinal stromal monitoring	Baseline FAPI uptake; on-treatment change; spatial immune readouts	FAPI uptake ≠ exclusion driver; requires immune/PD context

### 3.1 FAP-targeted “immune activation in place”

A major clinical trend is to use FAP as an address label to concentrate immune stimulation within CAF-rich stroma while limiting systemic exposure. One leading example is Simlukafusp Alfa (FAP-IL2v; RO6874281/RG7461), an anti-FAP antibody fused to an engineered IL-2 variant designed to bias signaling away from CD25 high cells and preferentially expand/activate effector populations in the tumor microenvironment (Verlingue et al., 2024; Prenen et al., 2024). Early clinical studies evaluated Simlukafusp Alfa alone and in combination with atezolizumab, providing human safety/tolerability and pharmacokinetic data and supporting the broader concept that FAP-directed cytokine delivery is feasible in advanced solid tumors (Koyama et al., 2024). For example, in a phase II esophageal cancer cohort, simlukafusp alfa plus atezolizumab achieved a disease control rate of 44.1%, including complete response in 2.9%, partial response in 17.6%, and stable disease in 23.5% of patients, illustrating early but still non-randomized clinical activity (Prenen et al., 2024).

A second, conceptually important approach is FAP-targeted costimulation, exemplified by RO7122290, a first-in-human, FAP-targeted split trimeric 4-1BB (CD137) ligand platform designed to enable costimulation preferentially in FAP^+^ tumor stroma and thereby reduce the systemic hepatotoxicity that historically limited some 4-1BB agonists (Melero et al., 2023). Together, these programs illustrate a “rewiring” logic: rather than depleting CAFs, they aim to override stromal immune gating by placing cytokine/costimulatory signals where excluded immune cells can be productively engaged.

### 3.2 CAF disruption and stromal ‘nodes’

Earlier clinical experience with sibrotuzumab (F19)—a humanized anti-FAP monoclonal antibody—demonstrated that repeated dosing in FAP^+^ cancers was feasible and generally well tolerated, but it also highlighted that binding FAP alone is rarely sufficient to remodel resistant niches without a payload, a co-target, or an ecosystem-informed combination strategy (Scott et al., 2003). To add functional potency, another clinical concept is to use FAP as an anchoring handle to cluster death signaling. RO6874813 (FAP-DR5 bispecific) was developed to bind FAP and conditionally cluster DR5 to promote apoptosis; early phase 1 experience has been reported in abstract form, offering a proof-of-concept for FAP-guided receptor agonism but also underscoring the need for clear pharmacodynamic readouts to confirm niche disruption in patients (Bendell et al., 2018).

### 3.3 Cellular immunotherapy against the stroma

Because FAP can be expressed in certain normal remodeling contexts, systemic stroma-directed cellular therapy raises on-target/off-tumor concerns. An important clinical mitigation strategy has been regional delivery. In the FAPME phase I trial, intrapleural delivery of autologous FAP-CAR-T cells in pleural mesothelioma was reported as safe, supporting the feasibility of locally deployed stroma-directed cellular immunotherapy and providing a clinically grounded template for “rewire-by-region” approaches (Hiltbrunner et al., 2021; Petrausch et al., 2012). This regional-delivery paradigm is relevant beyond mesothelioma because it aligns with the biology of immune exclusion: it can maximize exposure within a defined compartment and reduce systemic distribution, which is particularly attractive for targets expressed in activated fibroblasts.

### 3.4 Stratification and theranostics: FAP imaging as the front door for patient selection and monitoring

A practical challenge for CAF/FAP-directed therapy is that stromal abundance and spatial organization vary widely across tumors and patients. FAP-PET (FAPI tracers) has rapidly become a clinically useful strategy to (i) identify FAP-high tumors, (ii) quantify whole-body burden, and (iii) potentially monitor stromal dynamics on therapy (Liu et al., 2024; Mori et al., 2024). Notably, prospective clinical evidence has supported the diagnostic performance and safety of [^68^Ga]Ga-FAPI-46 PET as an imaging biomarker for detecting FAP-expressing tumors (Pabst et al., 2025). This imaging layer naturally enables theranostic translation (imaging-guided selection for FAP-targeted radioligand therapy). For example, clinical trials are evaluating agents such as ^177^Lu-FAP-2286 in advanced solid tumors, reflecting growing momentum toward an integrated “select → treat → verify” workflow centered on FAP biology (McConathy et al., 2022).

## 4 Safety and failure modes in FAP and CAF targeted niche rewiring

### 4.1 Target-biology risk: FAP is “tumor-enriched,” not tumor-exclusive

A central safety constraint is that FAP can be inducibly expressed outside tumors, especially in tissue remodeling contexts and in stromal populations that support normal physiology. In mouse models, systemic depletion of FAP^+^ stromal cells caused cachexia and anemia, highlighting that indiscriminate “CAF ablation” can damage supportive stromal compartments (Lavis et al., 2024). Similarly, potent cell-based targeting of FAP has shown dose-limiting bone toxicity and cachexia in preclinical settings due to recognition of FAP-expressing bone marrow stromal cells, emphasizing that “on-target/off-tumor” effects are a real design concern for cytotoxic modalities (Dong et al., 2025). Implication for this mini-review: the safest clinical logic is often rewiring (functional modulation) rather than wholesale depletion—especially when the modality can kill every FAP^+^ cell it contacts (Lee and Al-Ogaili, 2025). A second limitation is heterogeneity of FAP expression itself (Pleshkan et al., 2023). FAP abundance can differ substantially among tumor types, between primary and metastatic lesions, and even across lesions within the same patient. Moreover, FAP positivity does not define a single CAF state: FAP^+^ fibroblasts may overlap with matrix-remodeling, inflammatory, immune-interacting, or therapy-induced fibroblast programs depending on tumor context. Thus, FAP uptake or FAP immunoreactivity should not be equated automatically with a specific mechanism of immune exclusion. For example, a FAP-high tumor may be dominated by ECM stiffness, CXCL12-mediated chemokine gating, TGF-β signaling, or mixed stromal programs, each requiring a different therapeutic partner. This is why FAP-directed strategies should be integrated with orthogonal biomarkers, such as CAF-state signatures, spatial CD8 distribution, ECM/HA features, CXCL12–CXCR4 activity, TGF-β pathway activation, and myeloid composition.

### 4.2 Platform-specific liabilities: cytokines, costimulation, and radioligands have different “toxicity fingerprints”

FAP-targeted cytokines (FAP-IL2v/ Simlukafusp Alfa) aim to localize immune activation, but they still carry cytokine-class risks (hypotension/flu-like symptoms) and require exposure-aware dosing. Early clinical experience reported manageable safety with dose-limiting events such as hypotension at higher dose levels in a phase I setting (Koyama et al., 2024). Across phase II basket cohorts (such as cervical SCC; esophageal SCC), FAP-IL2v plus atezolizumab was described as tolerable, supporting feasibility while underscoring the need for biomarker enrichment and mechanism-aligned combinations to translate immune activation into durable benefit (Prenen et al., 2024; Hansen et al., 2024).

FAP-IL2v + pembrolizumab has also been explored clinically, with reports describing a manageable safety profile (even when efficacy signals were limited in certain settings), reinforcing that “localized cytokine delivery” is plausible but not automatically sufficient (Munoz-Couselo et al., 2025). For 4-1BB (CD137) agonism, historical hepatotoxicity with first-generation agonists established a clear class liability and motivated newer designs that restrict agonism to the tumor microenvironment (Khushalani et al., 2024; Leitner et al., 2023; Najjary et al., 2025). This is precisely where FAP-targeted 4-1BB agonists (RO7122290) fit: they are engineered to couple costimulation to FAP-rich stroma, aiming to preserve antitumor activity while reducing systemic liver inflammation associated with earlier CD137 agonists (FAP, 2023). For FAP theranostics/ radionuclide approaches, the safety logic shifts to dosimetry and off-target organ exposure. Contemporary imaging/theranostic reviews emphasize designing tracers with high tumor uptake, low off-target uptake, and rapid clearance to minimize toxicity, and they highlight the importance of careful dosimetric analysis as programs move into therapy (Lu-177–labeled FAP agents) (Mori et al., 2023). For rehabilitation-oriented care, safety monitoring should include brief functional screening (mobility, endurance, activities of daily living), because even ‘manageable’ immune or cytokine-class symptoms may translate into persistent impairment and reduced independence. This is particularly relevant as chronic immune-related adverse events after immunotherapy are increasingly recognized, supporting survivorship-style follow-up that tracks functional recovery alongside organ-based laboratory surveillance.

### 4.3 Failure modes: state mismatch, compensatory rebound, and insufficient pharmacodynamic verification

Even with acceptable safety, CAF/FAP programs can fail clinically if they target the wrong stromal mechanism for a given tumor state, or trigger compensatory pathways. These failure modes are consistent with broader lessons from stromal-targeting trials in PDAC. For example, pharmacologic Hedgehog/Smoothened inhibition was designed to reduce desmoplasia and improve drug delivery, but clinical studies did not establish meaningful benefit, and subsequent biological work suggested that some stromal programs can restrain tumor progression rather than simply support it. Together with HALO-301, these experiences indicate that “less stroma” is not necessarily equivalent to “better immunity” or “better drug delivery.” The more clinically relevant question is which stromal program is dominant, whether it is tumor-promoting or tumor-restraining in that context, and whether the intervention can shift the ecosystem toward productive immune infiltration without provoking compensatory rebound. Two recurring pitfalls are: i) State mismatch: treating a tumor that is not truly FAP-high/ stroma-dominant/ immune-excluded with a CAF-centric approach dilutes benefit. This is why FAP-PET (FAPI uptake) and stromal IHC are not optional but foundational for enrichment and interpretation (Mori et al., 2023). ii) Rebound programs: stromal modulation can be followed by myeloid recruitment, alternative fibroblast states, or renewed ECM remodeling, restoring exclusion. Practically, this argues for paired PD biopsies and spatial immune readouts (T-cell redistribution, myeloid shifts, ECM features) rather than relying only on radiographic response (Lee and Al-Ogaili, 2025). Thus, many negative stromal trials may represent failures of state selection, timing, combination design, or pharmacodynamic verification rather than simple failures of the stromal-targeting concept itself.

### 4.4 A pragmatic risk–mitigation checklist (what to pre-specify in trials)

Enrichment: baseline FAPI-PET high uptake and/or high FAP stromal IHC; plus a spatial phenotype consistent with immune exclusion (Mori et al., 2023). Mechanism-aligned combinations: cytokine delivery or costimulation plus checkpoint blockade, or stromal gating release plus ICB, chosen based on the dominant exclusion mechanism. Safety monitoring matched to modality: cytokine vital signs and vascular leak–like symptoms for IL-2 variants; LFT surveillance for CD137 biology; dosimetry/organ-at-risk tracking for radionuclides. Early PD endpoints: spatial T-cell penetration (margin to core), chemokine/cytokine shifts, CAF-state markers, and imaging changes in FAPI uptake to confirm that “rewiring” actually occurred (Mori et al., 2023).

## 5 From ecosystem-state diagnostics to a trial-ready roadmap for CAF/FAP niche rewiring

A practical trial framework should begin with baseline ecosystem classification rather than tumor histology alone. Candidate tumors should be evaluated for stromal burden, FAP expression, spatial immune phenotype, and the dominant exclusion mechanism, followed by a combination strategy selected to match that mechanism and early pharmacodynamic testing to confirm target engagement. In parallel, adding pragmatic rehabilitation endpoints (performance status, patient-reported physical function, and simple activity measures) can help distinguish biological niche engagement from meaningful patient benefit and can prompt timely referral to rehabilitation services during combination therapy.

Step 1-Select the right ecosystem state: Selection should begin with a stromal burden readout plus a spatial immune phenotype. For stromal burden, FAP-PET using FAPI tracers is increasingly positioned as a scalable, whole-body enrichment tool because uptake intensity can be linked to histologic FAP expression and helps identify “FAP-high” disease where stroma-directed delivery platforms have the most plausible therapeutic index. For immune phenotype, prioritize tumors with an “immune-excluded” architecture or strong stromal exclusion programs. The canonical example is the fibroblast/TGF-β–associated exclusion program that correlates with limited response to PD-L1 blockade and can be reversed in preclinical/human correlative settings when stromal signaling is addressed. Finally, incorporate CAF heterogeneity into enrichment: single-cell-informed CAF states (such as inflammatory vs. myofibroblastic CAF programs and immune-interacting CAF subsets) argue against “deplete-all” thinking and support stratifying by dominant CAF program (barrier-like vs. cytokine/chemokine-like vs. immune-modulatory). Importantly, FAP-high status should be considered an entry biomarker rather than a complete mechanism classifier; it should be interpreted together with CAF-state, immune-spatial, ECM, chemokine, and TGF-β-related readouts.

In this review, these endpoints are not proposed as stand-alone rehabilitation outcomes, but as pragmatic tolerability and implementation measures that complement oncologic endpoints by capturing whether stromal-rewiring combinations preserve treatment intensity, functional independence, and recovery during therapy.

Step 2-Match mechanism to dominant exclusion driver: Once “FAP-high/excluded” is confirmed, choose an intervention that directly targets the limiting step. If the main barrier is insufficient local immune activation, prioritize FAP-targeted immune activation in place, such as Simlukafusp Alfa (FAP-IL2v) or FAP-targeted conditional costimulation (RO7122290), which are designed to concentrate cytokine/costimulatory signals within FAP-rich stroma. If the dominant limiter is stromal gating (chemokine barriers, immune retention), use priming strategies that open trafficking pathways (CXCL12-axis disruption) before checkpoint blockade. If the dominant limiter is transport/pressure/ECM mechanics, consider barrier-relief programs that were explicitly tested with biomarker selection (such as HA-high PDAC with PEGPH20) as “lessons learned” for how strict enrichment and correlative endpoints must be built into trial design even when phase III outcomes are negative.
Step 3-Sequence and verify rewiring early: For immune-excluded tumors, sequencing is often the difference between biology and benefit. A common, testable hypothesis is prime→unlock→sustain: prime the stroma (barrier relief or chemokine gating release); unlock effector function with PD-L1 blockade; and sustain infiltration/function with localized immune activation (FAP-IL2v or conditional 4-1BB).

This mini-review emphasizes early pharmacodynamic endpoints that confirm niche rewiring: spatial CD8 redistribution (margin-to-core), changes in myeloid suppressive programs, and on-treatment shifts in stromal signatures; paired with noninvasive monitoring (serial FAPI-PET uptake dynamics) when feasible. For higher-risk modalities (cellular therapies), regional delivery illustrates a pragmatic safety-first approach that can still test the rewiring hypothesis in a constrained compartment (such as intrapleural FAP-CAR-T in mesothelioma). Taken together, the roadmap is simple but strict: enrich by state (FAP-high + excluded), treat with a mechanism-matched combination, and confirm early target engagement-otherwise CAF/FAP therapies will continue to be judged by late radiographic endpoints that do not reflect whether the ecosystem target was ever meaningfully engaged.
